# Applying visual mapping techniques to promote learning in community-based medical education activities

**DOI:** 10.1186/s12909-021-02646-3

**Published:** 2021-04-13

**Authors:** Sonali G. Choudhari, Abhay M. Gaidhane, Priti Desai, Tripti Srivastava, Vedprakash Mishra, Syed Quazi Zahiruddin

**Affiliations:** 1 Department of Community Medicine, Jawaharlal Nehru Medical College, Wardha, Maharashtra India; 2 School of Epidemiology & Public Health, Datta Meghe Institute of Medical Sciences, Wardha, Maharashtra India; 3 School of Health Professions Education & Research, Datta Meghe Institute of Medical Sciences, Wardha, Maharashtra India; 4 Department of Community Medicine, Jawaharlal Nehru Medical College, Wardha, Maharashtra India; 5 Faculty Development Committee, Association for Medical Education in Europe (AMEE), Maharashtra Wardha, India; 6 NMC Nodal Centre for National Faculty Development, Jawaharlal Nehru Medical College, Wardha, Maharashtra India; 7 Department of Physiology, Jawaharlal Nehru Medical College, Datta Meghe Institute of Medical Sciences (DU), Wardha, Maharashtra India; 8 Pro-Chancellor, Datta Meghe Institute of Medical Sciences, Maharashtra Wardha, India

**Keywords:** Visual mapping, Mind map, Concept map, Community-based medical education, Community medicine, teaching-learning, Rural

## Abstract

**Background:**

Teaching and learning Community-Based Medical Education (CBME) requires the active engagement of students in various activities to cover planned curricular content. CBME being multifaceted involves careful application and formation of links when attending to community health problems and public health issues. Students often depend on factual recall rather than ‘engaging in’, to counteract the broad and comprehensive nature of CBME. This study was conducted to assess the effectiveness of Visual mapping techniques as a learning tool in a CBME program for the subject Community Medicine and thereby assist medical undergraduate students in overcoming identified learning challenges.

**Methodology:**

An interventional study was conducted where medical undergraduates were randomly assigned to two different groups (each group = 30). After sensitization, a broad theme was taught to both the groups as a part of community-based teaching (CBT), each week for a month. The students in the intervention group were given the assignment to draw visual maps using the mind mapping & concept mapping techniques, after each CBT session, while the control group had Question-Answer session with built-in discussion (Conventional method) by an equally qualified, experienced faculty with no mapping assignments. A surprise written examination was conducted on the topics taught, and scores of both the groups were compared. Feedback was obtained from the intervention group.

**Results:**

Mean score of the examination by the intervention group (29.85 ± 3.22) was significantly higher than and that of the control group (23.06 ± 4.09) (t = 7.14 and *p* < 0.05). The students shared that the assignment of drawing mind and concept maps for topics taught helped in attempting questions of the written examination by facilitating easy recall of the information learned. It aided to frame the answers to descriptive questions in a structured way with the use of keywords. However, identifying the concepts and establishing relationship between them was slightly challenging.

**Conclusion:**

‘Visual mapping’ in the form of Mind and Concept mapping was found to be an effective learning tool for multifaceted CBME especially in promoting meaningful learning and facilitating rational thinking by the medical undergraduates.

## Background

Community-based teaching and learning is an important component of Community Based Medical Education (CBME), where students are supposed to understand people in their social context in a more holistic way [1]. Community based teaching (CBT) requires students to *listen*, *observe*, *organize*, *integrate*, and *correlate* different concepts and domains about health and disease epidemiology. They also need to acquire various clinical and public health skills etc. [2] It is essential for students to actively acquire the information, apply it and try to link different aspects of the community with the health of people residing there.

Students often fall short of such a broader and comprehensive approach while dealing with community health problems or public health issues. The majority of students yet depend on factual recall and are engaged in ‘*Rote learning’* which is easily forgotten. A negative consequence is that students may become unable to apply their knowledge to problem-solving situations or link this to previous knowledge [3, 4]. The subject Community Medicine or Preventive and Social Medicine is generally perceived by the students as challenging to conceptualize and retain. The possible reasons may include huge syllabus, variedness of content being a major subject, and inability to assemble, associate and integrate many concepts. Changing guidelines, strategies, and data concerning national health programs & policies add up to the learning difficulties.

Secondly, there are inbuilt multiple challenges and barriers for implementing CBT that may range from the de-novo learning environment, language barrier, adversities of the physical environment, scarce resources and clinical material, quality control..etc. in a community setting [5, 6]. The depth of learning from the CBME experience may be limited if students simply ‘arrive’ and never really ‘engage’ in a community. As per the Graduate Medical Education Regulations, 2018, an Indian Medical Graduate should function appropriately and effectively as a ‘*physician of first contact of the community’* while being globally relevant [7], and we feel that CBME does play a crucial role in the fulfilment of this competency.

A big challenge for medical educators is to search for pedagogical tools which will promote meaningful learning and discourage surface learning [8]. In the surface approach, the intention is only to cope with the task, and the learning resource material is seen as unrelated bits of information. This ultimately leads to much more restricted learning processes and encourages memorisation [4].

To overcome this challenge to some extent and to facilitate community based teaching-learning, in our study ‘*Visual mapping techniques’* were used as a learning tool for CBT.

‘Visual mapping’ is the technique used for displaying complex information visually. It is the graphical organization and presentation of information. Some of the types of visual maps are Mind maps, Concept maps, Conceptual diagrams, and Visual metaphors, etc. [9]

In our study, mind mapping and concept mapping were assessed as a learning tool for CBME for the subject of Community Medicine. The reason for choosing particularly these two mapping techniques is that the research on its use as a learning tool has been undertaken in health professions education including medical education in general as well as in specific subjects like Chiropractic education [10], Physical therapy [11], Anatomy [12], Biochemistry [13, 14], Pharmacology [15, 16], etc. However, it has been found that none of the ‘Visual mapping’ tools despite its potential suitability has been used for community-based medical education or in the context of community based teaching-learning. Hence, this study was carried out to study the effectiveness of identified visual mapping techniques i.e. Mind mapping and Concept mapping to promote learning in CBME activities for medical undergraduates.

## Objectives

The objectives of our study were to:

1. Sensitize the students about the use of ‘*Visual mapping techniques*’ including mind mapping and concept mapping, as a learning tool in Community based medical education.
2. Assess the effectiveness of mind mapping and concept mapping as a learning tool in CBME.
3. Explore the perception of the students regarding mind mapping and concept mapping as a learning tool in CBME.

## Methods

This interventional study was conducted at the department of Community Medicine and field practice area of Jawaharlal Nehru Medical College and Acharya Vinoba Bhave Rural Hospital, in Central India, from January 2018 to February 2019. Ethics approval to conduct the study was received from the Institutional Ethics Committee, Datta Meghe Institute of Medical Sciences (DU), Wardha (Maharashtra), India. The study participants were Final year (Part I) medical undergraduate students.

### Intervention

The intervention was the application of ‘Visual mapping’ techniques (Mind mapping and Concept mapping) as a learning tool for CBME of final year (Part-I) MBBS program for the subject Community Medicine.

### Description of mind mapping and concept mapping -

The core characteristics of these visual maps are rooted in the development of semantic networks, a 1950s’ technique for representing knowledge [17].

### Mind mapping

A ‘Mind map’ is a diagram used to represent words, ideas (in different colors, pictures) linked to and arranged around a central key topic or an idea. It is a learning tool that is simply a way to visualize a concept.

**Fig. 1:**
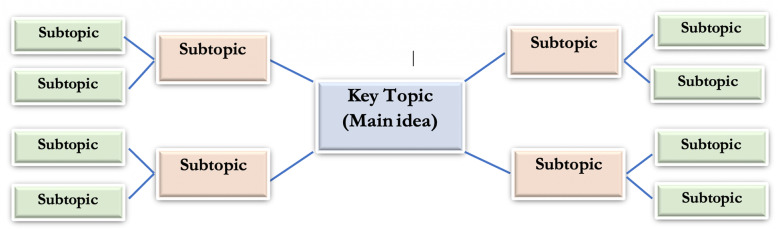
Basic structure of Mind map

Firstly the main study topic is drawn at the center, which allows the students 360 degrees of freedom to develop their mind map, with keywords, pictures branching at a divergent pattern. This represents different subtopics/categories, relevant to the main topic. From these main branches, sub-branches are created giving further details regarding the topic under study [18, 19]. (Fig. 1).

### Concept mapping

A concept map is a top-down diagram showing the relationships between concepts. It represents the systematic relationship or cross-connections among sub-concepts.

**Fig. 2:**
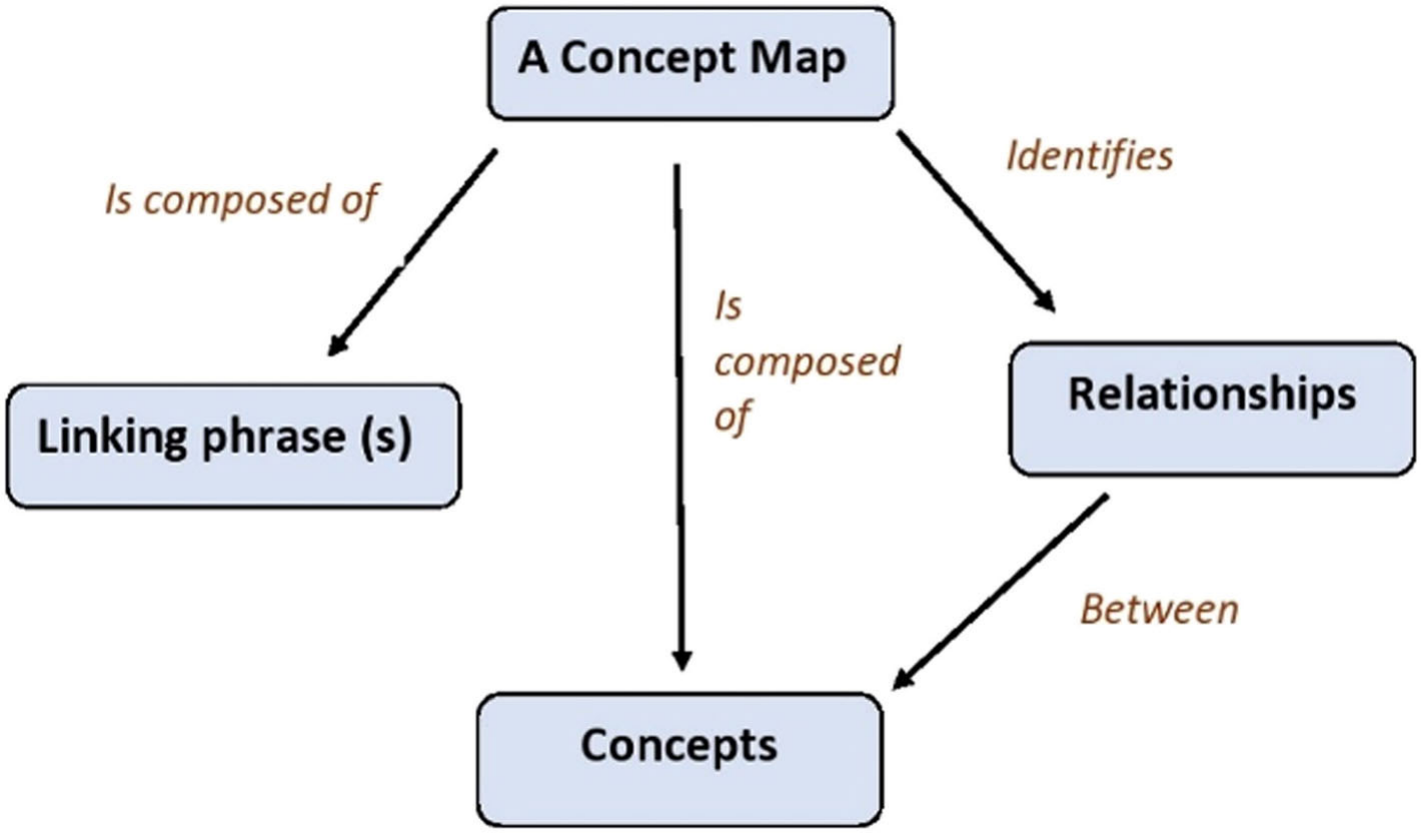
Concept Map showing key features of a concept map

It typically represents ideas and information as *boxes or circles*, which it connects with *labeled arrows* in a *downward-branching hierarchical structure*. Connector lines usually contain keywords or phrases that summarize the relationship between the topics they connect. Such as topic A “*causes*” topic B. Topics may be cross-linked with each other to depict more complex relationships. It usually contains general concepts at the top, with more specific concepts arrayed hierarchically below [20, 21]. (Fig. 2).

### Difference between mind mapping and concept mapping -

The major differentiating features between mind mapping and concept mapping are related to structuring. A mind map is centrifugal and more horizontal in structure with the study topic at the center and its details diverging peripherally while a concept map is a vertical or top-down diagram with the broad study topic at the top and more specific details as we go down. There is the use of linking phrases or keywords to depict the relationship between the concepts in a concept map unlike a mind map wherein there is a judicious use of colours, pictures. However, both mapping techniques promote learner’s active engagement.

### About community based medical education activities -

The said medical institute runs a flagship program termed ‘Comprehensive Community Health Care Program’ under which the medical students have the field or community visits to identified selected villages during their tenure as undergraduate. Students visit their allotted families in villages, build rapport, communicate and interact with the family members. Simultaneously they collect and record health-related information of the family members. Besides, community-based teaching and learning activities on pre-identified topics of public health significance (viz - Socio-cultural determinants of health, Infectious and Non-communicable disorders, Environment & Health, Housing, Water, Sanitation, Hygiene,.. etc) and observation of health days (viz- World health day, Population day, Environment day..etc) are carried out.

### Random allocation of study participants -

The whole study batch of final year (Part-I) medical undergraduate consisted of 200 students and two different selected villages were allotted to this batch for CBME activities. The students within the first hundred roll numbers (Batch A) were allotted a village named *Waifad.* While the remaining hundred students (Batch B) were assigned another village named *Lonsavali*.

Before the commencement of the study, a written informed consent explaining the purpose and seeking voluntary participation in the study was obtained from the students who expressed willingness to participate.

**Fig. 3:**
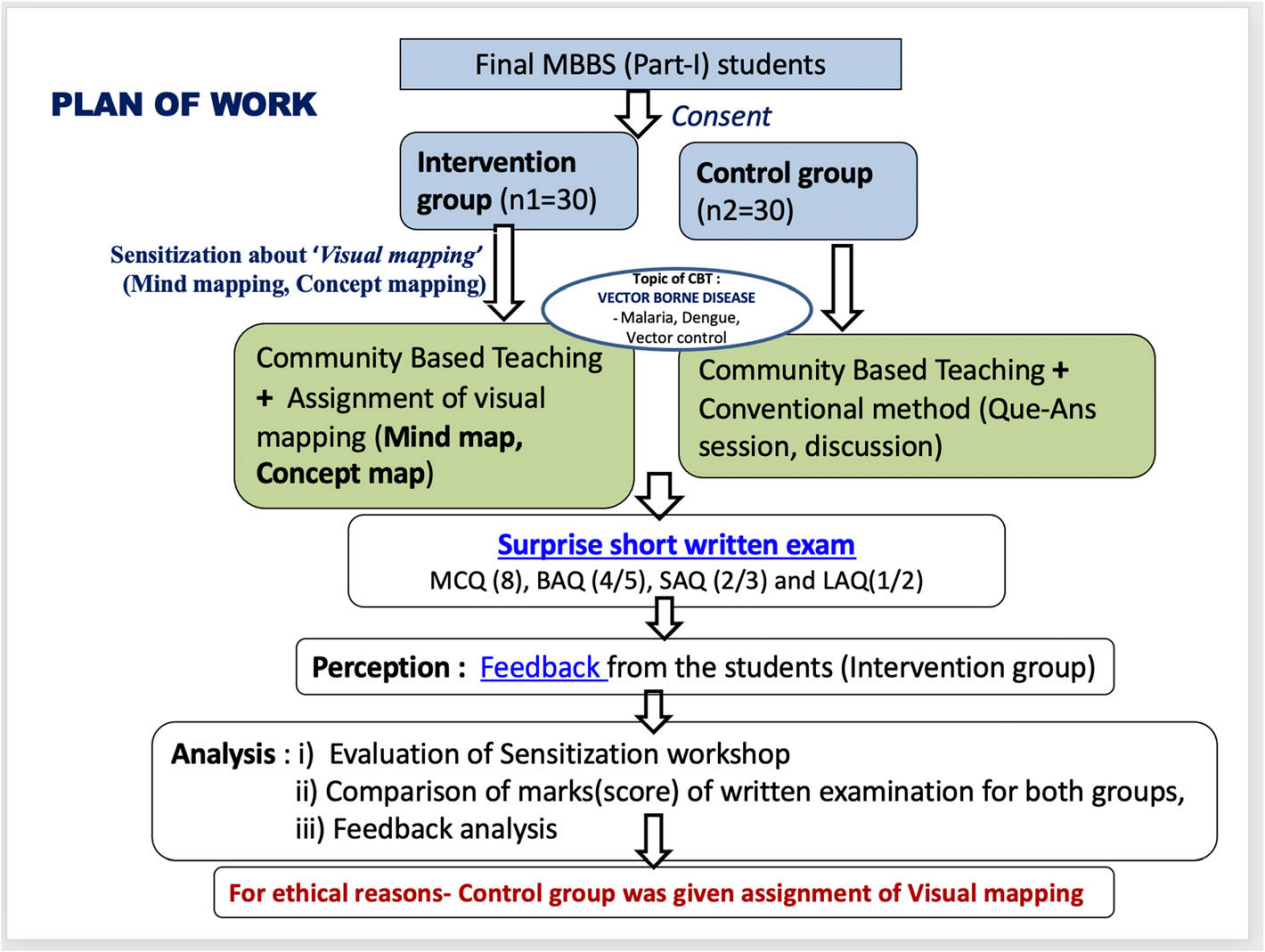
Group allocation and plan of work

Later, using a random number table, 30 students were sampled from ‘Batch A’ to form ‘Intervention Group’. Similarly from ‘Batch B’, another 30 students were randomly sampled to constitute ‘Control Group’. For the intervention group, the identified visual mapping techniques (i.e. Mind mapping and Concept mapping) were used as a learning tool for imparting CBME in their allotted village *Waifad* in comparison to another group that acted as a Control arm. (Fig. 3).

Both villages belonged to Seloo Block of Wardha District and the socio-economic, occupational, and cultural background of the communities in both the villages was similar. However, these two villages were distantly located and the community visits of the students were undertaken separately on different days of the week in an attempt to reduce the contamination bias.

### Data collection -

A mini-workshop was provided to sensitize the students of the intervention group to the proposed intervention i.e. identified visual mapping techniques. For this, two training sessions (two hours each, on two consecutive days) were undertaken by the investigator. The workshop started with a pre-test (score = 20) which consisted of both objective and descriptive questions on mind mapping, concept mapping, and CBME. In the first session, the students studied the purpose, method of drawing visual maps, and its relevance to the CBME. The method of creating maps was explained and demonstrated to students by simple examples. The second session included a hands-on experience during which students were asked to prepare the mind map and concept map of any topic based on their cognition. Their queries about these mapping techniques were resolved. The maps drawn were assessed on a 10-point rating scale (0 to 3 - Not satisfactory, 4 to 6 - Average and 7 to 10 - Satisfactory) and the post-test was similar to the pre-test.

Later as per the prescribed syllabus of community medicine for CBME, a broad topic/theme (*Vector-borne Diseases – Epidemiological determinants and clinical manifestations of vector borne diseases prevalent in India - like Malaria, Dengue; Concept of Integrated vector control including Mosquito control measures, Salient features of National Vector Borne Disease Control Program*) was taught to the students of both the groups during their field visits in respective villages as a part of CBT, each week for a month. The intervention group students were taught by the principal investigator and were given the assignment to draw visual maps using the mind mapping & concept mapping techniques after each CBT session for the subtopics taught. During each field visit, before the assigned CBT, the students presented the maps, elaborated on keywords, discussed possible doubts, and shared their experience of building the maps with their peers and faculty. Specific learning objectives were related to epidemiological determinants of vector-borne diseases, clinical features of prevalent diseases like Malaria, Dengue for early identification of suspected cases by frontline or grassroot level health care workers; community-based activities for prevention, and vector control. The faculty guided the students to correct their cognitive difficulties in organizing and clarifying the concepts and in removing queries if any.

The control group had the CBT followed by a question-answer session with a built-in discussion (conventional method) on a similar topic/theme by the equally qualified, experienced faculty with same set of learning objectives. As opposed to the intervention group, students in the control group were not required to submit a visual mapping assignment following their studies.

After the completion of thematic CBT sessions, for both the study groups, a surprise written examination (Theory) was conducted on the topics taught. The reason for undertaking the examination without notifying the students was to study the true effectiveness of the intervention on the performance of the students in the examination. The surprise mode did not give chance to the students to revise the topics taught in CBT sessions.

The question paper of the written examination consisted of questions; Multiple choice questions [8], Brief answer questions (4/5), Short answer questions (2/3), and Long answer questions (1/2). Around 40 to 50% of the questions were of higher cognitive level of Blooms’ taxonomy that included community/family case scenario or problem-based questions to determine the effectiveness of the intervention is not only the recall but also in concept building & critical thinking. The total allotted marks for the examination was 40.

The conduction of examination and assessment of the solved answer sheets of both the intervention and control group was carried out by a third party faculty of the same subject who was blinded for the intervention. This is done to nullify the investigators’ bias on students’ performance in the examination.

To assess the effectiveness of visual mapping techniques, later, the scores of written examination of both the intervention and control group were compared using the unpaired t-test for two sample groups. After the declaration of examination scores, perception regarding the use of the ‘Visual mapping technique’ was sought from the intervention group by administering a feedback questionnaire. The questionnaire consisted of a total of 18 questions including both close and open-ended questions. For some of the feedback questions, a five-point Likert scale, ranging from strongly disagree (score 1) to strongly agree (score 5) was incorporated.

**Study tools** used were:

i) Pretest and post-test questionnaire of the sensitization workshop
ii) Written examination question paper and
iii) Feedback proforma

### Statistical analysis

Data entry and analysis was done in Microsoft Excel and Stata (Version 12.0).

## Results

### Evaluation of the sensitization workshop

For the sensitization workshop, the attendance of the students in the intervention group was 100% i.e. 30/30. The mean pre-test score of the intervention group (n1 = 30) was 4.92 ± 2.47 out of the total maximum score of 20 and the post-test score was 16.03 ± 2.85. On using paired t test, a statistically significant difference was found between the mean scores at pre-test and post-test (t = − 14.64 and *p* < 0.05). The various parameters of cognitive learning gain were satisfactory. (Table 1) The demonstrable output of the sensitization activity was the mind maps and concept maps prepared by the students based on the information they had for a topic chosen by themselves. The maps were scored on a 10-point scale, wherein all the students got the score in the range of 7 to 10 (satisfactory).

**Table 1:** Parameters of cognitive learning gain for the students of the intervention group

Parameter	Formula	Value
Mean score of ‘gain in learning’	Mean post test score – Mean Pre test score	11.1
Absolute learning gain	{(Post test score- Pre test score)/Max score} × 100	55.55%
Relative learning gain	{(Post test score- Pre test score)/Pre test score} × 100	225.8%
Class average normalized gain(g)	Absolute gain (%)/max achievable gain (%)	(0.73) 73%
Average of single student normalized gain (gavg)	Summation of g of individual students/N	71.01%
Effect size	(Post test score - Pre test core)/ Average spread of Std deviation	4.13

### Analysis of written examination

The mean score of the written examination by the intervention group (n1 = 30) was 29.85 ± 3.22 out of a total maximum score of 40. The control group’s mean score was 23.06 ± 4.09. On using an unpaired t-test, a statistically significant difference was found between the two scores. (t = 7.14 and *p* < 0.05) (Table 2).

**Table 2:** Comparison of the mean scores of written examinations of two groups

Study groups	No.	Mean score of written examination (Maximum score = 40)	Std. deviation	Std Error of mean	Confidence interval of the difference	t- score	df	*P* value
Intervention group	n1 = 30	29.85	3.22	0.587	4.8876 to 8.6924	7.14	58	< 0.05 (significant)
Control group	n2 = 30	23.06	4.09	0.746				

### Feedback analysis

The feedback about the use of ‘Visual mapping’ techniques (Mind mapping and Concept mapping) as a learning tool, was obtained from the students of the intervention group (n1 = 30). The majority of students (27, 90%) who provided feedback reported a lack of knowledge of these mapping techniques before this activity; however, 3 students had come across these mapping terminologies before. However, all the students of the intervention group reported that they used mind mapping and concept mapping for the first time. The maximum number of students (23, 76.6%) took an average of 20 to 30 min to develop a single map.

Four students (13.3%) knew about the availability of software for making the visual map online. The examples they shared were –*mindmaple, freemind, mindmup2, C map, Coggle*. Three students (10%) were aware of other mapping techniques like Vee mapping, Visual metaphor, etc.

**Fig. 4:**
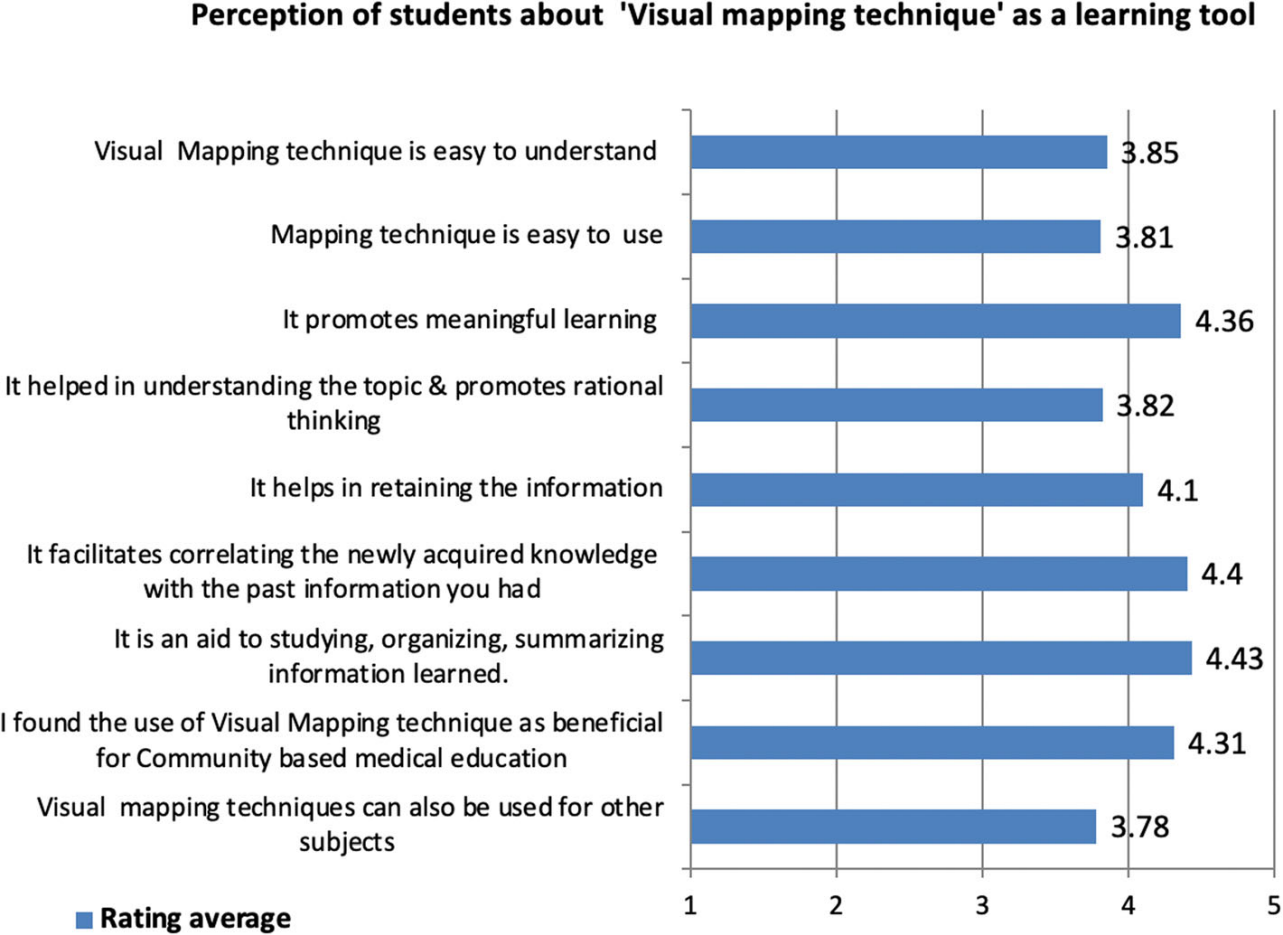
Feedback analysis

For the parameters rated on a five-point Likert scale, ranging from strongly disagree (score 1) to strongly agree (score 5), the analysis was done by calculating ‘*Rating average’*. The maximum score i.e. 4.43 was obtained for ‘*It is an aid to studying, organizing, summarizing information learned*’ followed by ‘*It facilitates correlating the newly acquired knowledge with the past information you had’* (4.40), *‘It promotes meaningful learning* (4.36), etc. (Fig. 4).

All students in the intervention group reported that the task of drawing the mind maps and concept maps helped them in answering questions of the unplanned written test. Various remarks by the students to open-ended questions about how the assignment of visual maps benefitted them were as follows:

i. *“The maps helped overall by facilitating the easy recall of the information learned.”*
ii. *“Visual maps aided to frame the answers of the descriptive questions in a structured way”.*
iii. *“We realized the importance of headings, subheadings, and keywords for a topic and successfully incorporated them in the answer.”*
iv. *“We could write the answers along with examples.”*

The strengths of visual maps as mentioned by the student were - Visual mapping promoted active & self-directed learning and rational thinking too. It makes the learning more enjoyable & interesting by revealing one’s thought process.

The key suggestions by the participants were - Visual mapping especially concept mapping should also be used by the teachers as a teaching modality for explaining the complex concepts during didactic lecture; To maximize the utility of visual map for learning, the mapping assignment must be followed by a group discussion on the created maps.

However, few of the students encountered certain challenges while using these maps. These challenges included students running out of space while creating hand-drawn maps on paper. Identifying the concepts and establish the relationship between them was a chaotic experience for some of the students. The exercise was perceived as a time-consuming task by a few.

*Concept maps in particular* were more difficult to prepare than mind maps as it requires a good understanding of individual concepts, links, and how they overlap with other concepts as a prerequisite.

**Fig. 5:**
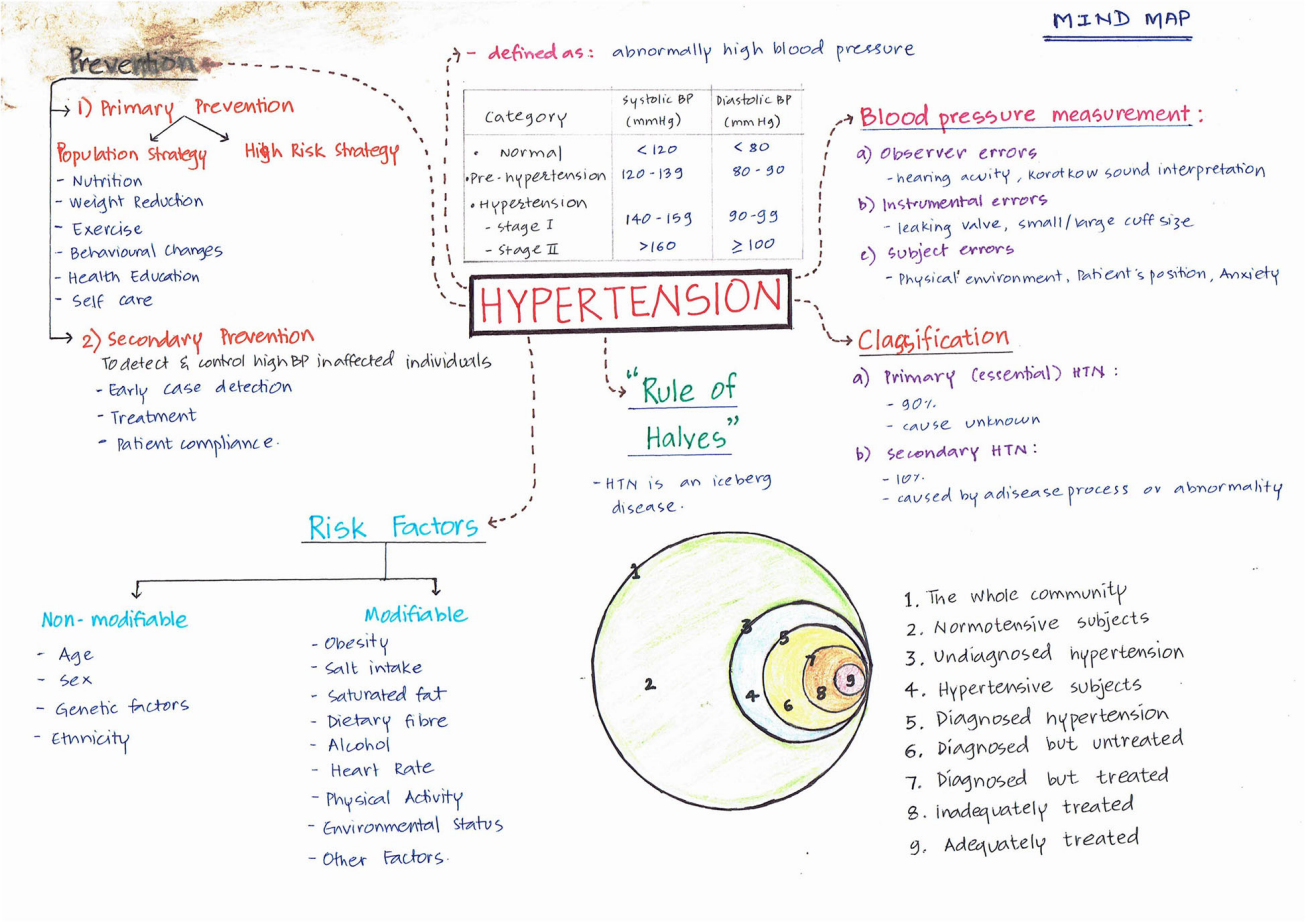
Mindmap on Hypertension

**Fig. 6:**
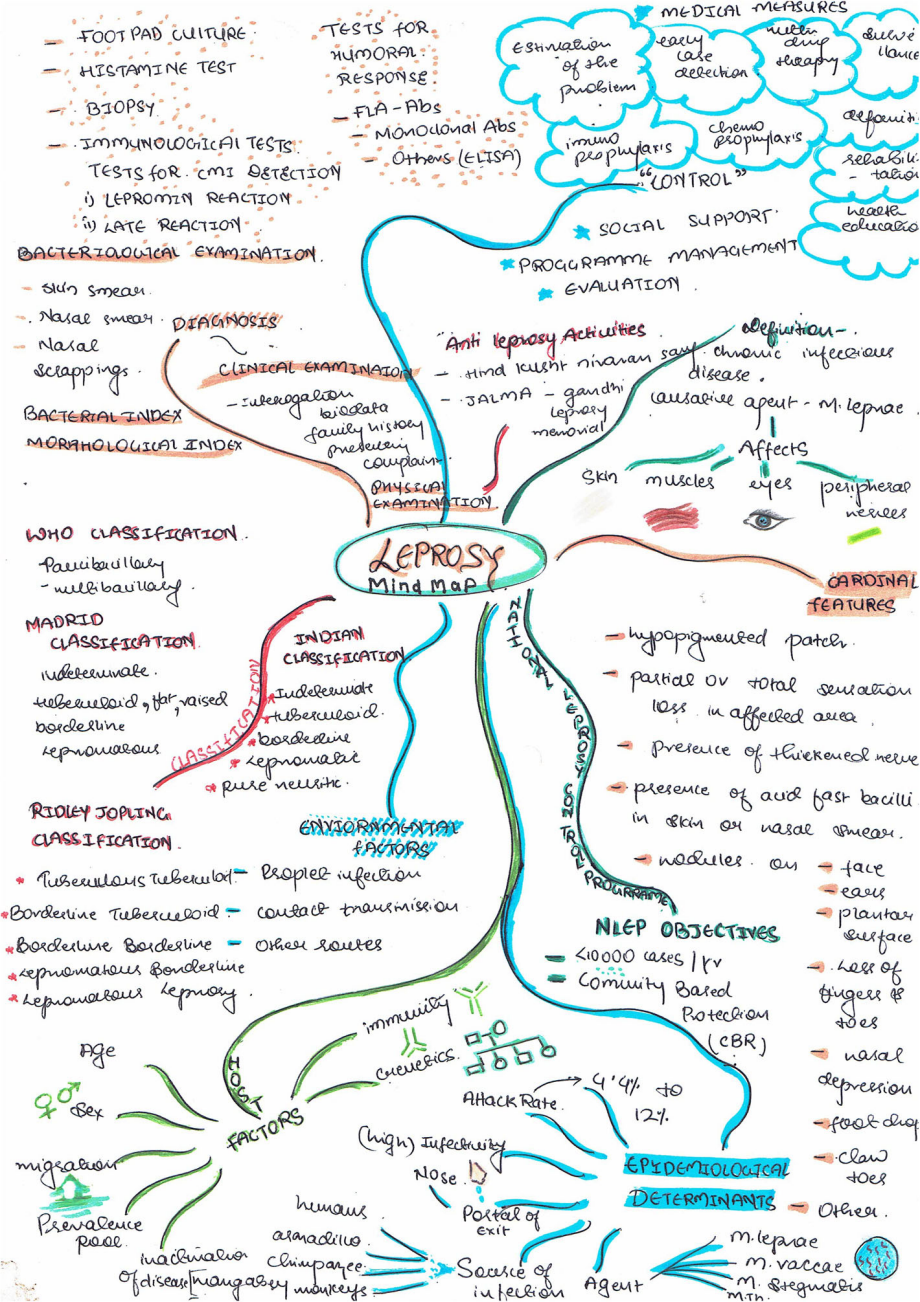
Mindmap on Leprosy

**Fig. 7:**
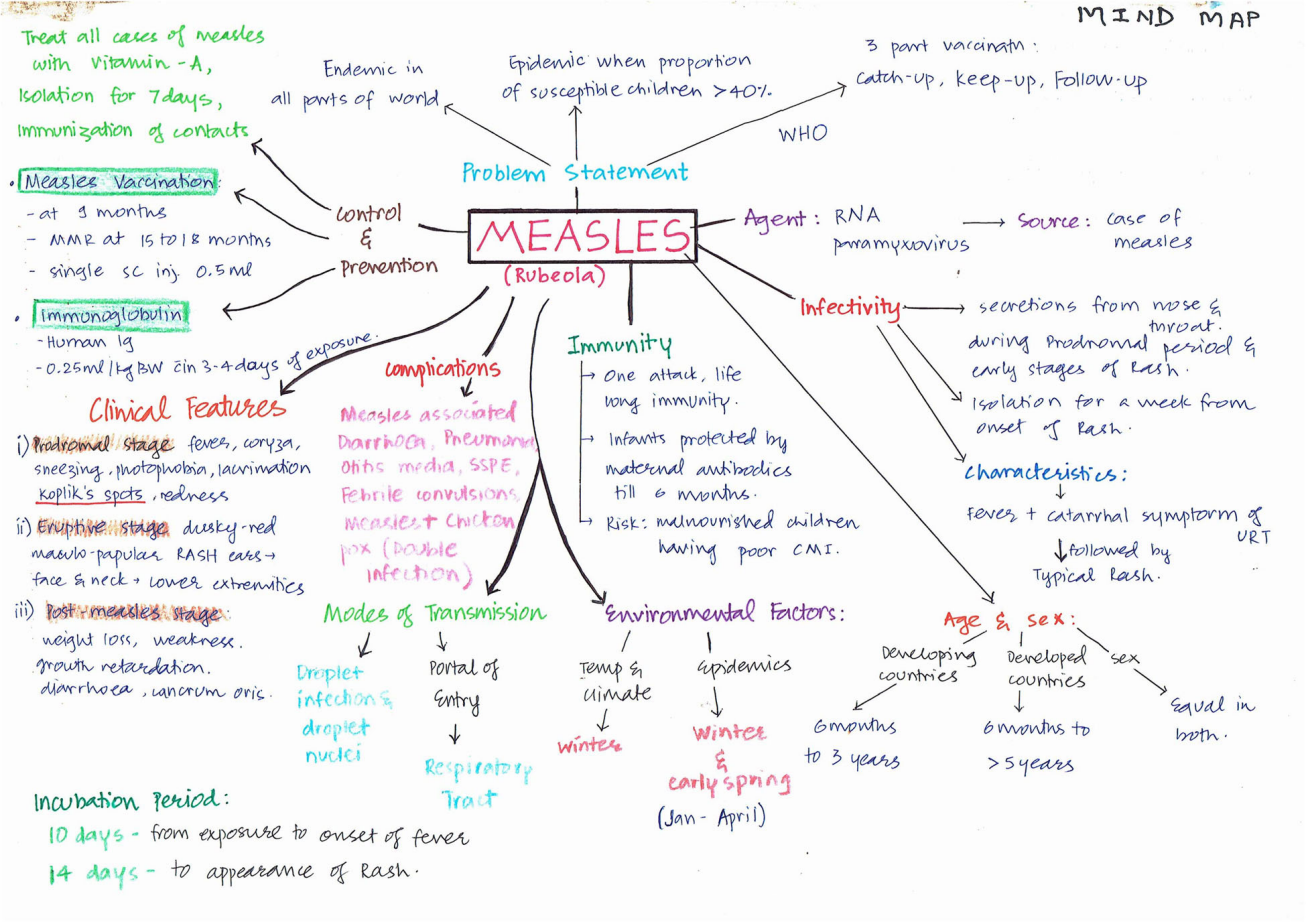
Mindmap on Measles

**Fig. 8:**
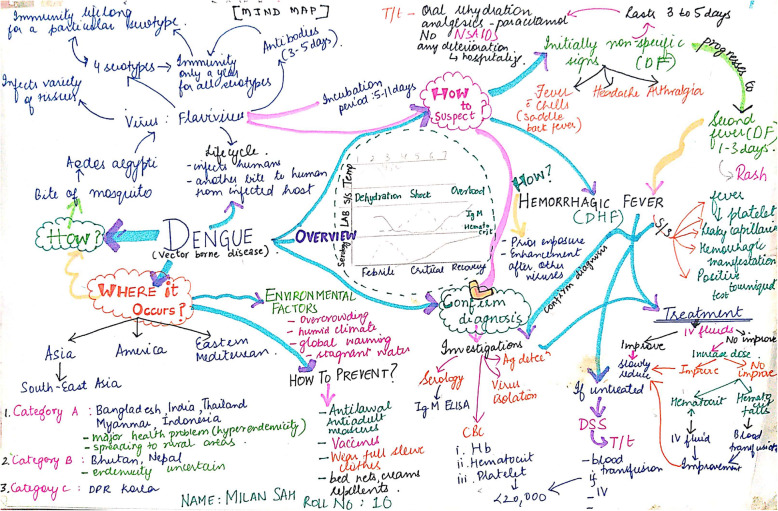
Mindmap on Dengue

**Fig. 9:**
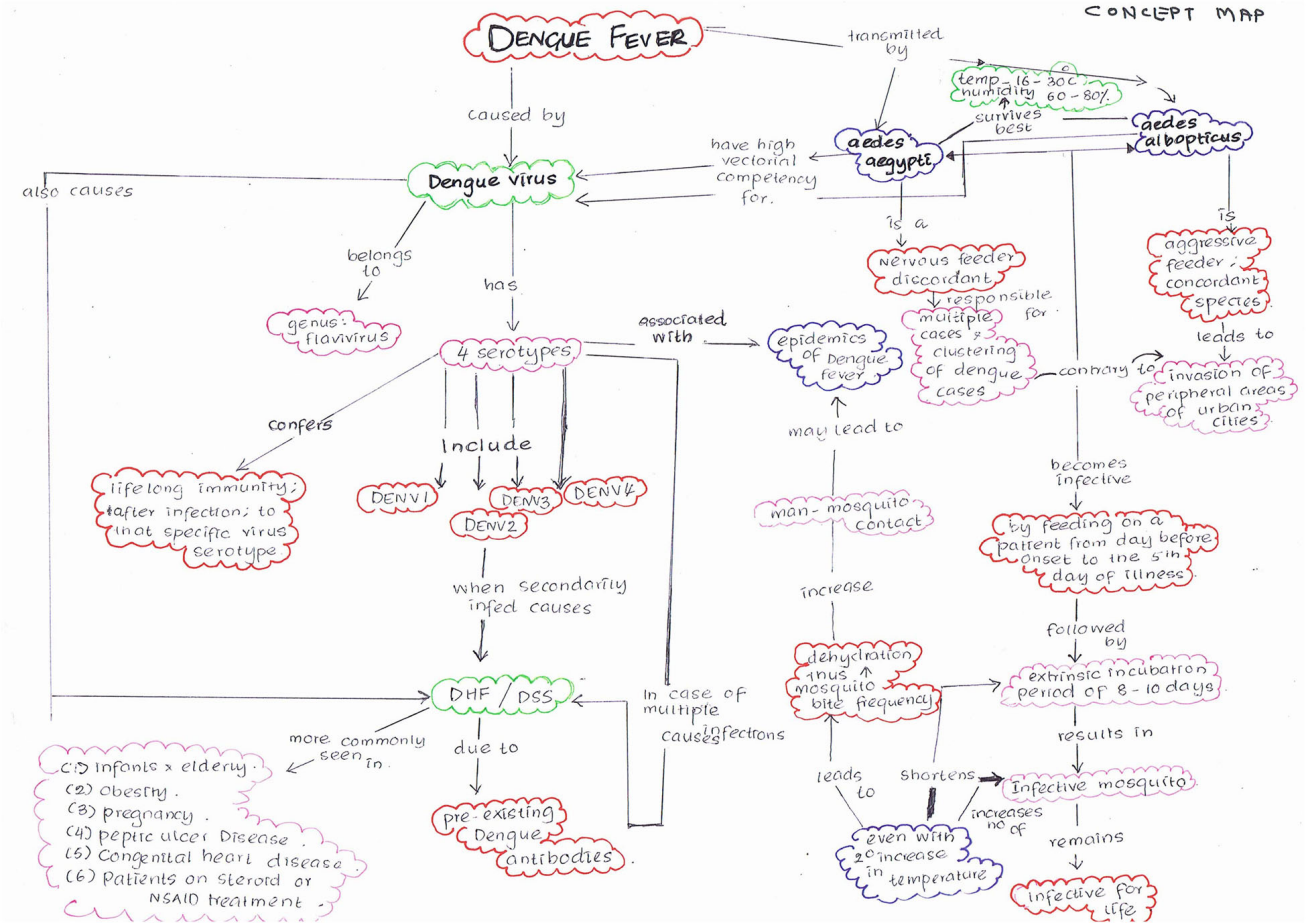
Concept map on Dengue

**Fig. 10:**
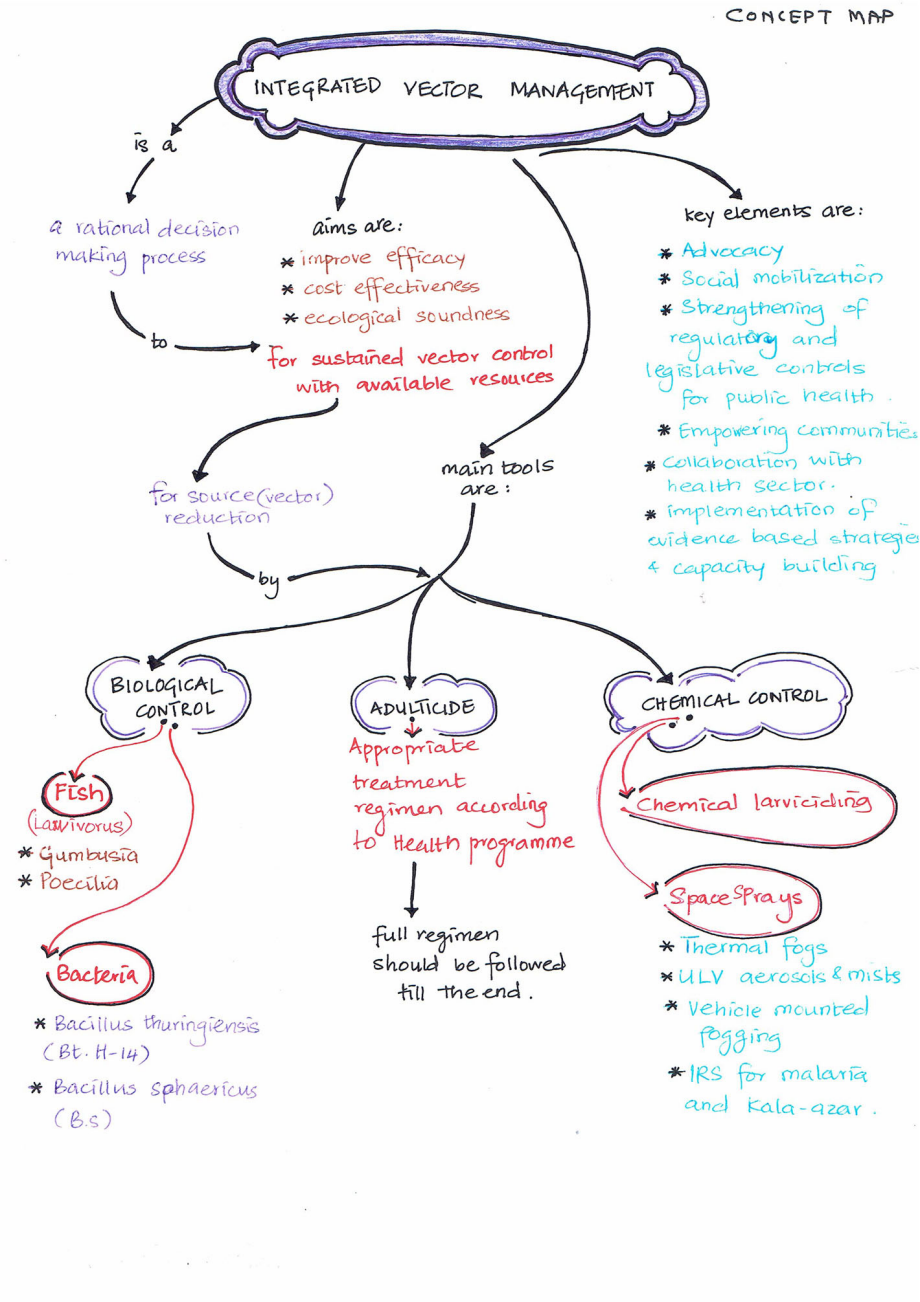
Concept map on Integrated Vector Control

Visual maps (Mind maps and Concept maps) were prepared by the students during sensitisation workshop and as a part of the assignment after each CBT session taught. Few of the maps are depicted (Figs. 5, 6, 7, 8, 9 and 10). Drawing a map is a creative activity and it varies from student to student. It is challenging to create a perfect map considering the technicalities of drawing it. However, the whole exercise of map-making was worth it as a good and a unique learning experience.

## Discussion

Community-based teaching-learning activities are carried out in an open rural setting that is free from the boundaries of four walls of a typical classroom. Here the students get the opportunity to observe and listen much more than what is taught. They can explore many ideas, concepts using the natural setting of field practice areas [22]. However, unlike didactic lectures in the classroom, CBT is usually not a tightly structured and time-bound activity. There is always some degree of flexibility concerning teaching-learning processes when it takes place in a community setting taking into consideration various barriers for undertaking such activity [23].

Learning is a complex cognitive process that occurs in individuals of all ages. ‘*Meaningful learning’* requires an understanding of the various topics and concepts of the subject under study. Learning with understanding allows integration of new concepts with previously learned concepts and leads to retention of information in long-term memory in a usable manner [24].

In our study, two specific visual mapping techniques i.e. mind mapping and concept mapping was used as a learning tool by the medical undergraduates in the context of CBME. Here, the students of the intervention group were oriented to these mapping techniques by conducting a training workshop. This sensitization activity was evaluated by a three-pronged strategy viz. checking the students’ attendance, pre-test, and post-test analysis and by ensuring the preparation of visual maps (mind maps, concept maps) as per the instructions given to them. In concurrence with this, Sumanbala et al [16*]* included a two-hour session of concept mapping using pre-prepared concept maps on general awareness and pharmacotherapy of HIV and AIDS. Similarly, in the study by Farida Quadir et al [15*],* for test groups, two introductory sessions were arranged for orienting students to concept mapping. Deshatty D and Mokashi V [12], provided the participants of the mind map group, two training sessions for how to develop and apply the mind map in the best way. During training, participants were allowed to ask queries regarding the technique used. However, these studies [12, 15, 16] have no mention of the evaluation of such training activity. Our study emphasizes the need for mandatory evaluation of such sensitization sessions, as the satisfactory training of the study participants is the prerequisite for the successful application of intervention in the future.

A significant difference was observed in the mean score of the written examination given by the students of intervention (29.85 ± 3.22) and control group (23.06 ± 4.09) (*p* < 0.05), in our study. This is in line with a Deshatty et al [12*]* study which reported that students using mind maps in studying Anatomy scored better than the ‘standard note taking’ group, though the difference was not statistically significant. However, the passing percentage of students was 38% from the mind map group compared to only 26% students from the other group. Similarly, in a study by Ali Bawaneh [25], it was noticed that the mean score of the mind map group was higher than the conventional group both for immediate achievement and retention. (*p* < 0.05). This is also in line with Farida Quadir et al [14] which revealed that for best choice questions as well as for short essay questions, there was no statistically significant difference. Similarly, Amila Wickramasinghe et al [26*]* stated that the average marks obtained by the mind map group was 31.3% and it was 37.6% in the self-selected study technique group with no statistically significant difference. In the context of concept mapping, *Krishna M. Surapaneni and Ara Tekian* [13] *studied the utility of concept mapping for learning Biochemistry* where the first-year medical students (*n* = 150) were randomly divided into two groups of 75. One group attended the traditional program and the other the innovative program. Students in the innovative program using concept mapping outperformed those in the traditional didactic program in the written knowledge tests.

In our study, the feedback from the participants in the intervention group favoured the utility of the visual maps as a learning tool. The majority of the students opined that these maps were like aid to studying, organizing, summarizing information learned and it promoted active & meaningful learning. They also stated that the assignment of visual mapping helped in attempting questions of the written test by facilitating an easy recall of the information learned. This is supported by similar findings from Deshatty D et al [12] where the majority of students who used mind mapping in Anatomy perceived that it helped them in learning and encouraged its use as a learning tool in gross Anatomy along with the standard note-taking method. Along the same line, Wang S et al [27*]* applied a mind map in teaching and learning activities of Medical Immunology and noted an improvement amongst learners in logically correlating various knowledge points and overall depiction. He further stated about the utility of mind mapping in promotion of divergent thinking and building innovative approaches amongst users. As a learning modality, it promotes creativity, facilitates retention by memory, and develops problem-solving ability amongst learners [28]. In the study by *Krishna M. Surapaneni and Ara Tekian* [13], *t*he students evaluated the relevance of the learning process using a questionnaire, where they gave a high positive rating for the innovative course with concept mapping (93–100% agreement). Besides, Amila Wickramasinghe et al [26], stated that the majority of students (97.1%, *N* = 34) from the mind map group felt that it is useful to summarize information and 87.9% wanted to study further mind mapping. Similarly, the study by Farida Qadir et al [15*]* explored the use of concept mapping as a facilitative tool to promote learning in Pharmacolog*y* and showed that the technique helped the students to conceptualize difficult topics in CNS pharmacology (86.36%). The study mentioned that the concept mapping was particularly beneficial in preparing for examination as it provided a quick overview of the entire subject (68.68%).

Amongst the literature on visual mapping techniques, an article by Martin J Eppler [29] was found to be one of the comprehensive and standard references for use of visual mapping strategies. Eppler compared the concept mapping technique to three other types of visualization formats, namely mind maps, conceptual diagrams, and visual metaphors. The application parameters and the respective assets and pitfalls of each format for learning and knowledge sharing were reviewed and discussed. It is argued that the combination of these four visualization types can play to the strength of each one. The article provides real-life examples for such use in undergraduate and graduate university teaching. It recommends that the different visualization formats can be used in complementary ways to enhance motivation, attention, understanding, and recall.

In a nutshell, CBT can be described as the type of educational activity, where the students employ activities and are usually given an overview of the topics that are based on the needs of the society, community, and family and relevant to the local area. The learning objectives are multiple covering many different areas of the subject [30]. Thus there is plenty of room for self-directed and experiential learning by students after attending CBT activities and observing the physical, social, biological environment of the community and family. In such a scenario, it can be stated that mind mapping and concept mapping can help medical students, with their efforts towards meaningful learning in CBME.

### Limitation of study

To study the role of ‘Visual mapping’ in retention, periodic and surprise assessments of the students for the same topic could have been done. However, due to time constraints, such follow-up student assessment was not undertaken, in this study. Again due to feasibility issues, the ‘*Learning style* and *approach’* of the individual student was not assessed and/or considered for analysing the utility of visual mapping as a learning tool, though these factors may affect the receipt of these mapping techniques as a learning tool. Additional limitations such as relatively small sample size, the possibility of contamination bias in case the students of the intervention group might have shared their mapping experience with the control arm, cannot be denied.

### Recommendation

Visual mapping techniques should be used and assessed as a ‘Teaching’ as well as student ‘Assessment’ tool. Secondly, the effectiveness of mind mapping, concept mapping as a learning tool can also be determined for other subjects & disciplines in health sciences with a larger sample size. Apart from these two techniques, other visual mappings like the *Conceptual diagram, Cognitive maps, Visual metaphor, Vee mapping*, etc. should also be used and further assessed for their utility.

## Conclusion

Use of ‘Visual mapping techniques’ in the form of Mind mapping and Concept mapping as a learning tool for CBME in the subject of Community Medicine, for the final year MBBS (Part-I) students, revealed that it was effective in promoting meaningful learning and in facilitating rational thinking by the students, as depicted from a higher score in written examination and the feedback.

## Data Availability

The datasets used and/or analysed during the current study are available from the corresponding author on reasonable request.

## Abbreviations

CBME: Community based medical education
CBT: Community based teaching
MBBS: Bachelor of Medicine and Bachelor of Surgery
